# Learning semi-supervised enrichment of longitudinal imaging-genetic data for improved prediction of cognitive decline

**DOI:** 10.1186/s12911-024-02455-w

**Published:** 2024-05-28

**Authors:** Hoon Seo, Lodewijk Brand, Hua Wang

**Affiliations:** Department of Computer Science, Colorado School of Mines, Golden, Colorado 80401 USA

**Keywords:** Alzheimer’s disease, Multi-modal, Longitudinal learning, Enrichment

## Abstract

**Background:**

Alzheimer’s Disease (AD) is a progressive memory disorder that causes irreversible cognitive decline. Given that there is currently no cure, it is critical to detect AD in its early stage during the disease progression. Recently, many statistical learning methods have been presented to identify cognitive decline with temporal data, but few of these methods integrate heterogeneous phenotype and genetic information together to improve the accuracy of prediction. In addition, many of these models are often unable to handle incomplete temporal data; this often manifests itself in the removal of records to ensure consistency in the number of records across participants.

**Results:**

To address these issues, in this work we propose a novel approach to integrate the genetic data and the longitudinal phenotype data to learn a fixed-length “enriched” biomarker representation derived from the temporal heterogeneous neuroimaging records. Armed with this enriched representation, as a fixed-length vector per participant, conventional machine learning models can be used to predict clinical outcomes associated with AD.

**Conclusion:**

The proposed method shows improved prediction performance when applied to data derived from Alzheimer’s Disease Neruoimaging Initiative cohort. In addition, our approach can be easily interpreted to allow for the identification and validation of biomarkers associated with cognitive decline.

## Background

Alzheimer’s disease (AD) is a neurodegernative condition in which people suffer from the progressive deterioration of cognitive functions, such as memory, language, and judgment. The World Health Organization (WHO) predicts that AD will affect 75 million people by 2030 and 132 million people by 2050 [1]. To address this major public health challenge, it is critical to detect AD at an early stage from both the therapeutic and research standpoints. Recent works [2, 3] have analyzed the progression of AD through modeling and predicting clinical assessments. Furthermore, in the last decade [3], rich neuroimaging measurements, such as magnetic resonance imaging (MRI), have been widely used to predict the clinical outcomes associated with AD.

Despite these efforts, many existing approaches [3, 4] suffer from the following limitations. First, because a lot of models routinely carry out the learning tasks at each time point of the AD progression separately, they cannot leveage the temporal relationships across the longitudinal records. Given that AD is a progressive neurodegenerative disorder, multiple consecutive records should be analyzed together for keeping track of the disease progression. Therefore, it is ideal that modern statistical learning techniques can study the temporal variations in the records that are consistent with how we expect a progressive disease to behave. Second, temporal records are often missing at certain time points, which results in an inconsistent number of records per participant. This make it difficult to apply traditional statistical methods that work in the setting when the data at all time points provided. Third, current longitudinal methods [4, 5] tend to focus on measurements derived from MRI scans, such as FreeSurfer (FS) and voxel-based morphometry (VBM), rather than genotype information, such as single-nucleotide polymorphisms (SNPs). It is known [6] that genetic factors can be strong predictors of future cognitive decline, therefore it is important to integrate longitudinal phenotype measurements with genetic data that remain constant when AD develops. Finally, the clinical outcomes of participants assessed from cognitive ability tests, such as Ray’s Auditory Verbal Learning Test (RAVLT), are often provided in resources such as the Alzheimer’s Disease Neuroimaging Initiative (ADNI), which can be used as data labels for better predicting a future AD diagnosis. Therefore it is of great interest to explore how to use such labeled data to learn data representations with improved predictive power.

In an attempt to overcome the first limitation and uncover the temporal structure of brain phenotypes, several longitudinal prediction models [7, 8] have been proposed. However, these models represent the temporal imaging records as a tensor, which inevitably increase the complexity of the prediction problem and require that each participant has the same number of temporal observations. Since each participant must have the same number of observations, the user of these approaches must discard samples that have a number of records below a given threshold, which may potentially lose valuable information of the input data. Other approaches [9, 10] have relied on imputation techniques to estimate the missing records. Yet these imputation methods may incur undesirable artifacts, which may introduce biases into the final predictions of the longitudinal models.

To handle the longitudinal multi-modal prediction problem with incomplete temporal neuroimaging records, in this work we propose a semi-supervised learning method to learn participant-specific projections to enrich the multi-modal phenotypic measurements, which is an extension of our earlier short conference paper [11]. We analyze the consecutive imaging records simultaneously and learn a projection for each participant. To take advantage of temporal and modality relationships, we introduce trace-norm regularization over the concatenation of all participant-specific projections to maintain their global consistency. Furthermore, a structured sparsity-induced norm regularization is applied to learn the group-structured representations of genetic data, which are integrated with the enriched representations for imaging data. Finally, our model factorizes the enriched biomarker representations, available clinical scores, and genetic biomarkers of participants with the common participants representations. The aim of these factorizations is to extract the representations for a participant shared across the different modalities. As a result, the learned projections from imaging data are tightly coupled with the genetic modality and available clinical scores. Provided with the learned projections per-participant, we can transform the multi-modal representations extracted from phenotypes with varied data sizes and the measurements of the genetic biomarkers into an enriched biomarker representation with a fixed length. With a fixed-length vector per participant, we can freely make use of conventional machine learning models to predict clinical scores associated with AD.

## Methods

In this section, first we will formalize the problem to learn a fixed-length biomarker representation for each participant. Then we will then gradually develop our learning objective. Finally, an efficient computational algorithm will be derived to solve our proposed objective.

### Notations and problem formalization

Throughout this paper, we write matrices as bold uppercase letters and vectors as bold lowercase letters. The *i*-th row, the *j*-th column, and the element at *i*-th row and *j*-th column of the matrix $\textbf{M} = \left[ m^i_j \right]$ are denoted as $\textbf{m}^i$, $\textbf{m}_j$, $m^i_j$, or $\textbf{e}_i^T \textbf{M}$, $\textbf{M} \textbf{e}_j$, $\textbf{e}_i^T \textbf{M} \textbf{e}_j$, respectively, where we define $\textbf{e}_j$ as the *j*-th column of the identity matrix $\textbf{I}$. When $p \ge 1$, the $\ell _p$-norm of a vector $\textbf{v} \in \Re ^d$ is defined as $\left\| \textbf{v} \right\| _p = \left( \sum _{i=1}^d v_i^p \right) ^p$. For a matrix $\textbf{ M }$, the trace of $\textbf{ M }$ is defined as $\text {tr}( \textbf{M} ) = \sum _{ i } m^i_{i}$. The Frobenius norm of $\textbf{M}$ is defined as $\left\| \textbf{M} \right\| _{F} = \sqrt{\sum _{i=1}^n \sum _{j=1}^m |m^i_{j}|^2}$. The $\ell _{ 2,1 }$-norm of $\textbf{ M }$ is defined as $\Vert \textbf{M}\Vert _{2, 1} = \sum _{i=1}^{n} \sqrt{\sum _{j=1}^{m}\left| m^i_{j}\right| ^{2}} = \sum _{i=1}^{n}\left\| \textbf{m}^{i}\right\| _{2}$. The trace norm of $\textbf{M}$ is defined as $\left\| \textbf{M} \right\| _{*} = \sum _{i=1}^{min\{n,m\}} \sigma _i$, where $\sigma _i$ is the *i*-th singular value of $\textbf{M}$.

**Fig. 1:**
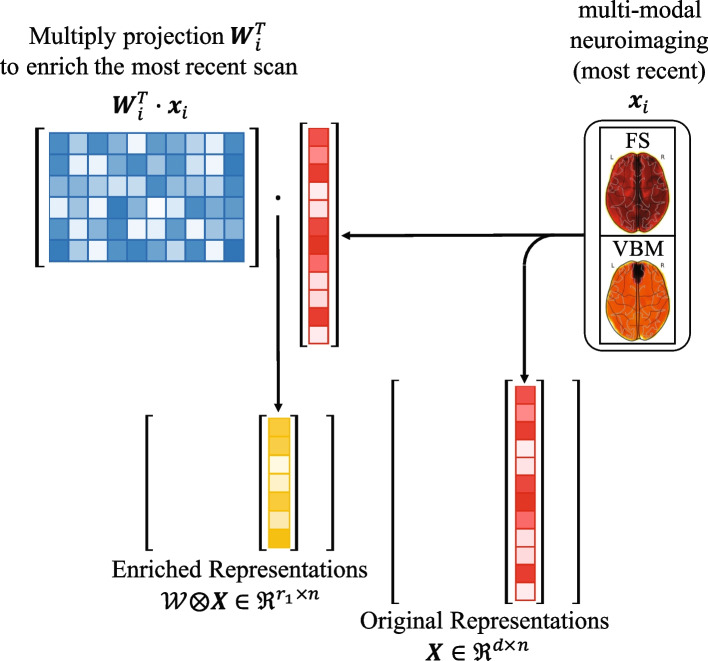
Illustration of original and enriched biomarker representations. The goal of the enrichment model is to learn the set of projections $\mathcal {W}$ and project the last record. As a result, the dimensionality of enriched representation $r_1$ is much smaller than the dimensionality of original representation *d*

Given a neuroimaging dataset, phenotypic measurements are usually described by the biomarkers extracted from brain scans. Mathematically, the medical records of the *i*-th participant in a studied cohort can be denoted as $\mathbf {\mathcal {X}}_i = \{\textbf{X}_i, \textbf{x}_i\}$, where $i\ =\ 1,2,\cdots ,n$ indicates the index of participant. Here, $\textbf{X}_i\ =\ [\textbf{x}_{i1},\cdots ,\textbf{x}_{in_i}]\in \Re ^{d \times n_i}$ collects the available medical records of the *i*-th participant from the baseline (first time point) to the second last visit, such that the total number of the medical records of the *i*-th participant is $n_i$ + 1. We note that $n_i$ varies across the dataset due to inconsistent/missing temporal records of the participants. We use $\textbf{x}_i \in \Re ^d$ to denote the last medical record of the *i*-th participant and use $\textbf{X}\ =\ [\textbf{x}_1,\cdots ,\textbf{x}_n ]$ to summarize these records of all the participants in the studied cohort. Because multiple types of biomarkers, such as VBM and FS markers, can be extracted from the set of brain scans, we concatenate the vector representations of these biomarkers as the phenotypic assessment of a participant. For example, in our study we write $\textbf{x}_{ij}=[\textbf{x}_{ij}^{VBM}, \textbf{x}_{ij}^{FS}]$ and $\textbf{x}_i=[\textbf{x}_i^{VBM}, \textbf{x}_i^{FS}]$, where $1 \le i \le n,\ 1 \le j \le n_i$. Because $\{\textbf{x}_{ij}\}_{j=1}^{n_i}$, together with $\textbf{x}_{i}$, describe the temporal changes of the phenotypes of the *i*-th participant over time, $\mathcal {X}_i$ is a summarization of the ***dynamic*** measurements of the *i*-th participant, which is also broadly called as the longitudinal measurements in the literature of medical image computing [4, 7, 8, 12, 13]. To make use of $\textbf{X}_i$ and $\textbf{x}_i$ together, we can use longitudinal enrichment to learn a fixed-length vector from them [14–17]. Specically, we learn a projection tensor $\mathcal {W} = \{\textbf{W}_1, \textbf{W}_2, \cdots , \textbf{W}_n\} \in \Re ^{d \times r_1 \times n}$, by which we can compute the fixed-length biomarker representations $\mathcal {W}^T \otimes \textbf{X} = [\textbf{W}^T_1 \textbf{x}_1, \textbf{W}^T_2 \textbf{x}_2, \cdots , \textbf{W}^T_n \textbf{x}_n] \in \Re ^{r_1 \times n}$ for the entire cohort, *i.e.*, we project $\textbf{x}_i$ by $\textbf{W}_i$ for the *i*-th participant by computing $\textbf{z}_i = \textbf{W}_i^T \textbf{x}_i \in \Re ^{r_1}$. A schematic illustration of the projected (enriched) biomarker representations is shown in Fig. 1.

In addition to the phenotypic measurements used in a neuroimaging data set, genotypes of the same cohort may also be available, such as the SNP profiles of the participants, that can be represented by $\textbf{X}_{SNP}$ = $[\textbf{x}_1^{SNP}, \cdots , \textbf{x}_n^{SNP}] \in \Re ^{d_{SNP} \times n}$, where $\textbf{x}_i^{SNP}$ is the vector representation of the SNP profile of the *i*-th participant. Here we note that $\textbf{X}_{SNP}$ is ***static*** that does not vary over time when AD develops.

Besides the input phenotypic (dynamic) and genotypic (static) data, the outputs of the prediction tasks are cognitive status of the participants, which are usually assessed by the clinical scores of a set of cognitive tests. We use $\textbf{Y}_l \in \Re ^{c \times l}$ to list the clinical scores of the first *l* participants at her or his last visit, where *c* is the total number of clinical scores in studied in our work. Here, without losing generality we consider the first *l* samples as the labeled data for training. Apparently, $\textbf{Y}_l$ can be used as the labeled data to enable us to learn the data representation with supervision, which could potentially improve the predictive power of the learned data representations.

In the following subsection, we will develop our learning objective gradually.

### Our objective

We start by learning the representations of the static genetic data to utilize the group structures of SNPs [18, 19]. Recent developments in high-throughput genotyping techniques allow new methods to investigate the effect on brain structures and functions of genetic variation. Many previous association studies treated the SNPs as independent units and ignored underlying relationships between the units. However, multiple SNPs from the same gene are naturally related so that such SNPs often jointly perform the genetic functionalities together. To incorporate the group structures associated with SNPs, we propose to learn the representations of the genetic data of a studied cohort by minimizing the following objective:

1 $$\begin{aligned} \mathcal {J}_0(\textbf{H}_0, \textbf{G}_0)\ =\ \left\| \textbf{X}_{SNP} - \textbf{H}_0 \textbf{G}_0 \right\| _{2,1} + \alpha \left\| \textbf{H}_0 \right\| _{G_{2,1}}. \end{aligned}$$

In Eq. (1), the first term factorizes $\textbf{X}_{SNP}$ into $\textbf{H}_0$ and $\textbf{G}_0$, where $\textbf{H}_0$ can be seen as the compressed view of the SNP features [20] and $\textbf{G}_0$ describes the new representations of the *n* participants in the subspace spanned by $\textbf{H}_0$ [21]. To find the group structure of SNPs, we leverage the linkage disequilibrium (LD) [22] which defines the non-random association between alleles at various loci. Then we capture the group-wise sparsity in $\textbf{H}_0$ by making use of the group $\ell _{2,1}$-norm ($G_{2,1}$-norm) regularization term $\left\| \textbf{H}_0 \right\| _{G_{2,1}} = \sum _{k=1}^K \left\| \textbf{H}_0^k \right\| _{2,1}$, where $\textbf{H}_0 = [\textbf{H}_0^1; \textbf{H}_0^2, \cdots ; \textbf{H}_0^K]$ consists of *K* groups derived from the LD correlations of the SNPs [18, 19]. Here we choose to use the $\ell _{2,1}$-norm distances to improve the robustness of our model against outliers [23–26].

Next we study how to learn a vector representations with fixed length for every participant from their image data in varied sizes. While the genetic profiles of the participants remain constant over time, the functions and structures of the brains of the participants change as AD progresses. Therefore AD progression is characterized by the longitudinal imaging records extracted from the multiple brain scans that change over time. However, the longitudinal imaging records pose a critical challenge to build the predictive models, because different participants may take the brain scans at different time and the number of brain scans of different participants are not same in general. To deal with this difficulty and summarize the brain variations of every participant individually, we propose to learn a vector representation with the fixed length from the image data of each participant $\{\textbf{X}_i, \textbf{x}_i \}$ with the varied size $n_i$ by computing $\textbf{z}_i = \textbf{W}_i^T \textbf{x}_i \in \Re ^{r_1}$.

First, to preserve as much dynamic information of $\textbf{X}_i$ as possible, we propose to learn the projection $\textbf{W}_i$ for the *i*-th participant by minimizing the following objective of the principal component analysis (PCA) [27]:

2 $$\begin{aligned} \mathcal {J}_1(\textbf{W}_i)\ =\ \left\| \textbf{X}_i - \textbf{W}_i \textbf{W}_i^T \textbf{X}_i \right\| _{2,1},\quad s.t.\ \textbf{W}_i^T \textbf{W}_i = \textbf{I}. \end{aligned}$$

Here again we use $\ell _{2,1}$-norm objective in the PCA to enhance the robustness of the learned projection $\textbf{W}_i$ against outlying samples which is unavoidable in the large dataset [23–26].

Second, besides using the projection learned from each individual participant separately, to maximize the consistency across all the learned projections for the same cohort, we enforce the low-rank consistencies onto the learned projection matrices by introducing two trace-norm regularization terms as following [7, 13, 17]:

3 $$\begin{aligned}&\mathcal {J}_2(\mathcal {W})\ =\ \sum _{ i = 1 }^{ n } \left\| \textbf{ X }_{ i } - \textbf{ W }_i \textbf{ W }_i^ { T } \textbf{ X }_{ i } \right\| _{2, 1} + \beta \left( \left\| \textbf{W}_{(1)} \right\| _{*} + \left\| \textbf{W}_{(2)} \right\| _{*} \right) ,\nonumber \\&\quad s.t.\ \textbf{ W }_i^{ T } \textbf{ W }_i = \textbf{ I }, \end{aligned}$$

where $\textbf{W}_{(1)} = \left[ \textbf{W}_1, \textbf{W}_2, \cdots , \textbf{W}_n \right] \in \Re ^{d \times (r_1 \times n)}$ and $\textbf{W}_{(2)} = \left[ \textbf{W}_1^{T}, \textbf{W}_2^{T}, \cdots , \textbf{W}_n^{T} \right] \in \Re ^{ r_1 \times (d \times n)}$ are two unfolded matrices of the local projection tensor $\mathcal {W}$.

Finally, equipped with the learned representations for imaging features in multiple modalities and genetic features, we integrate them together to explore the full potential of an imaging-genetic dataset. First, we write the temporally enriched representations for image data together as $\textbf{Z} = \left[ \textbf{z}_1, \dots , \textbf{z}_n\right] = \mathcal {W}^T \otimes \textbf{X} \in \Re ^{r_1 \times n}$. Following the same idea as before, we factorize $\textbf{Z}$ and align the factorized data representation with that learned from the static genetic data $\textbf{G}_0$ by minizing the following objective:

4 $$\begin{aligned} \mathcal {J}_3&(\textbf{H}_0, \textbf{H}_1, \textbf{G}_0, \textbf{G}_1, \mathcal {W}) = \gamma _1 \sum _{ i = 1 }^{ n } \left\| \textbf{ X }_{ i } - \textbf{ W }_i \textbf{ W }_i^ { T } \textbf{ X }_{ i } \right\| _{2, 1}\nonumber \\&+ \gamma _2 \left\| \mathcal {W}^T \otimes \textbf{X} - \textbf{H}_1 \textbf{G}_1 \right\| _{2, 1} + \gamma _3 \left\| \textbf{X}_{ \text {SNP}} - \textbf{H}_0\textbf{G}_0 \right\| _{2, 1} + \gamma _4 \left\| \textbf{G}_1 - \textbf{G}_0 \right\| _{2, 1}\nonumber \\&+ \gamma _5 \left\| \textbf{H}_0 \right\| _{G_{2}} + \gamma _6 \left( \left\| \textbf{W}_{(1)} \right\| _{*} + \left\| \textbf{W}_{(2)} \right\| _{*} \right) + \gamma _7 \left\| \textbf{U} \right\| _{1}, \quad s.t. \quad \textbf{W}_i^{ T } \textbf{ W }_i = \textbf{I}, \end{aligned}$$

where $\gamma _1, \gamma _2, \cdots , \gamma _7$ are hyperparameters of our learning model.

Now we can perform the association studies between the clinical scores and the new data representations learned from our model. Suppose that the clinical scores of $\textbf{Y}_l \in \Re ^{c \times l}$ are obtained in *c* cognitive assessments for the *l* training samples, we use $\textbf{F} = [\textbf{F}_l, \textbf{F}_u] \in \Re ^{c \times n}$ to denote our estimated clinical scores and use the constraint $\textbf{F}_l = \textbf{Y}_l$ to make use of the training data $\textbf{Y}_l$, by which we can conduct the regression analyses by minimizing the following objective:

5 $$\begin{aligned} \mathcal {J}&(\textbf{U}, \textbf{F}, \textbf{H}_0, \textbf{H}_1, \textbf{G}_0, \textbf{G}_1, \mathcal {W}) = \left\| \textbf{F} - \textbf{U}^T \textbf{G}_1 \right\| _{2, 1} + \gamma _1 \sum _{ i = 1 }^{ n } \left\| \textbf{ X }_{ i } - \textbf{ W }_i \textbf{ W }_i^ { T } \textbf{ X }_{ i } \right\| _{2, 1}\nonumber \\&+ \gamma _2 \left\| \mathcal {W}^T \otimes \textbf{X} - \textbf{H}_1 \textbf{G}_1 \right\| _{2, 1} + \gamma _3 \left\| \textbf{X}_{ \text {SNP}} - \textbf{H}_0\textbf{G}_0 \right\| _{2, 1} + \gamma _4 \left\| \textbf{G}_1 - \textbf{G}_0 \right\| _{2, 1}\nonumber \\&+ \gamma _5 \left\| \textbf{H}_0 \right\| _{G_{2}} + \gamma _6 \left( \left\| \textbf{W}_{(1)} \right\| _{*} + \left\| \textbf{W}_{(2)} \right\| _{*} \right) + \gamma _7 \left\| \textbf{U} \right\| _{1},\nonumber \\&\quad s.t. \quad \textbf{F}_l =\textbf{Y}_l, \ \textbf{W}_i^{ T } \textbf{ W }_i = \textbf{I}. \end{aligned}$$

**Fig. 2:**
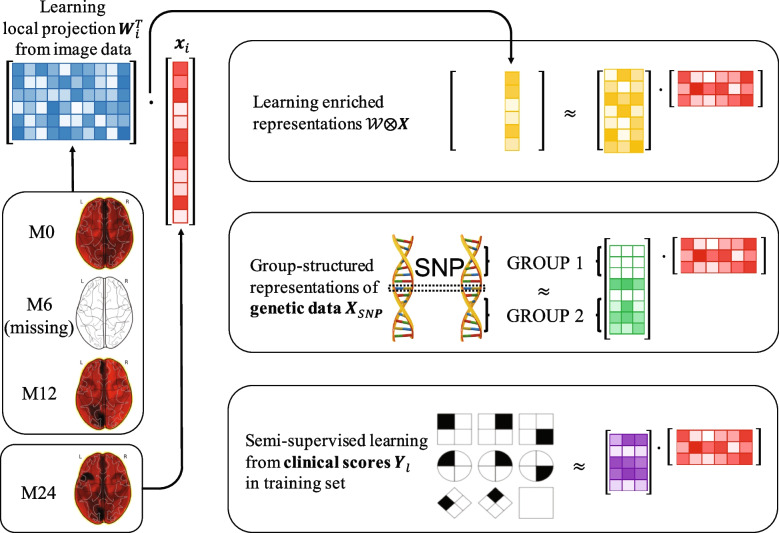
Overview of proposed semi-supervised learning framework to fully utilize the potential of a longitudinal AD dataset. We use factorization to extract the common representations of participants shared across genetic, image, and clinical scores data. As a result, the genetic and clinical scores data can be reflected in the learned projections $\mathcal {W}$

Our new method is schematically illustrated in Fig. 2.

### The solution algorithm

Although our objective in Eq. (5) has clearly motivated, it is difficult to solve in general, because it is non-smooth. Thus in this subsection we drive an efficient solution to optimize our objective. Using the optimization framework presented in the earlier work [28, 29] that proposed the iterative reweighted method to solve non-smooth objectives, we can solve Eq. (5) by an iterative procedure (Algorithm 1 in [28]) in which the key step is to minimize the following objective:

6 $$\begin{aligned}&\mathcal {J}^{R}(\textbf{U}, \textbf{F}, \textbf{H}_0, \textbf{H}_1, \textbf{G}_0, \textbf{G}_1, \mathcal {W}) = \text {tr}((\textbf{U}^{T}\textbf{G}_{1} - \textbf{F})^{T}\textbf{D}_{1}(\textbf{U}^{T}\textbf{G}_{1} - \textbf{F}))\nonumber \\&+ \gamma _1\sum _{i=1}^{n}\text {tr}((\textbf{X}_i-\textbf{W}_i\textbf{W}_i^T\textbf{X}_i)^T\textbf{D}_{2,i}(\textbf{X}_{i}-\textbf{W}_i\textbf{W}_i^T\textbf{X}_i))\nonumber \\&+\gamma _2\text {tr}((\mathcal {W}^T\otimes \textbf{X}-\textbf{H}_1\textbf{G}_1)^T\textbf{D}_3(\mathcal {W}^T\otimes \textbf{X}-\textbf{H}_1\textbf{G}_1))\nonumber \\&+ \gamma _3\text {tr}((\textbf{X}_{SNP}-\textbf{H}_0\textbf{G}_0)^T\textbf{D}_4(\textbf{X}_{SNP}-\textbf{H}_0\textbf{G}_0))\nonumber \\&+\gamma _4\text {tr}((\textbf{G}_1 - \textbf{G}_0)^T\textbf{D}_5(\textbf{G}_1 - \textbf{G}_0)) +\gamma _5\text {tr}(\textbf{H}_0^T\textbf{D}_6\textbf{H}_0)\nonumber \\&+\gamma _6\text {tr}(\textbf{W}^T_{(1)}\textbf{D}_7\textbf{W}_{(1)}) +\gamma _6\text {tr}(\textbf{W}^T_{(2)}\textbf{D}_8\textbf{W}_{(2)}) +\gamma _7\sum ^{c}_{q=1}(\textbf{u}_q^T\textbf{D}_{9,q}\textbf{u}_q),\nonumber \\&\quad s.t. \quad \textbf{F}_l =\textbf{Y}_l, \ \textbf{ W }_i^{ T } \textbf{ W }_i = \textbf{ I }, \end{aligned}$$

where $\textbf{D}_1, \textbf{D}_{2,i}, \textbf{D}_3, \textbf{D}_4, \textbf{D}_5, \textbf{D}_{9,q}$ are diagonal matrices whose *j*-th diagonal element $d_*^j$ of $\textbf{D}_*$ is as follows:

7 $$\begin{aligned}&d_1^j = \frac{1}{2 \sqrt{\left\| \textbf{e}_j^T(\textbf{U}^T\textbf{G}_1-\textbf{F})\right\| ^2_2+\delta }},d_{2,i}^j = \frac{1}{2 \sqrt{\left\| \textbf{e}_j^T(\textbf{X}_i-\textbf{W}_i\textbf{W}_i^T\textbf{X}_i)\right\| ^2_2+\delta }},\nonumber \\&d_3^j= \frac{1}{2 \sqrt{\left\| \textbf{e}_j^T(\mathcal {W}^T\otimes \textbf{X}-\textbf{H}_1\textbf{G}_1)\right\| ^2_2+\delta }},d_4^j = \frac{1}{2 \sqrt{\left\| \textbf{e}_j^T(\textbf{X}_{SNP}-\textbf{H}_0\textbf{G}_0)\right\| ^2_2+\delta }},\nonumber \\&d_5^j = \frac{1}{2 \sqrt{\left\| \textbf{e}_j^T(\textbf{G}_1 - \textbf{G}_0)\right\| ^2_2+\delta }}, d_{9,q}^j = \frac{1}{2 \sqrt{(u^j_q)^2+\delta }},\nonumber \\&\textbf{D}_8 = \frac{1}{2}(\textbf{W}_{(2)} \textbf{W}_{(2)}^T + \delta \textbf{I})^{- \frac{1}{2}}, \textbf{D}_7 = \frac{1}{2}(\textbf{W}_{(1)} \textbf{W}_{(1)}^T + \delta \textbf{I})^{- \frac{1}{2}}. \end{aligned}$$

$\textbf{D}_6$ is a block diagonal matrix, where *k*-th block (*k*-th group of SNPs) is $\frac{1}{2}(\left\| \textbf{H}_0^k\right\| ^2_F+\delta )^{-\frac{1}{2}}\textbf{I}_k$. $\textbf{I}_k\in \Re ^{d_{k}\times d_{k}}$ is a identity matrix, and $d_k$ denotes the number of rows (the number of SNPs) of *k*-th block of $\textbf{H}_0=[\textbf{H}_0^1;\textbf{H}_0^2;\cdots ;\textbf{H}_0^K]$, so that $\sum _{k=1}^K d_k = d_{SNP}$. The dimensions of the matrices in Eq. (7) are: $\textbf{D}_1 \in \Re ^{c \times c},\ \textbf{D}_{2,i} \in \Re ^{d \times d},\ \textbf{D}_3 \in \Re ^{r_1 \times r_1},\ \textbf{D}_4 \in \Re ^{d_{SNP} \times d_{SNP}},\ \textbf{D}_5 \in \Re ^{r_2 \times r_2},\ \textbf{D}_6 \in \Re ^{d_{SNP} \times d_{SNP}},\ \textbf{D}_7 \in \Re ^{d \times d},\ \textbf{D}_8 \in \Re ^{r_1 \times r_1},\ \textbf{D}_{9,i} \in \Re ^{r_2 \times r_2}$.

To minimize the smoothed objective Eq. (6), we use the Alternating Direction Method of Multipliers (ADMM), which is proposed in [30, 31]. By introducing two more constraints $\textbf{A} = \textbf{U}$ and $\textbf{B}_{(2)} = \textbf{W}_{(2)} \Leftrightarrow \textbf{B}_{i} = \textbf{W}_{i}$ ($i=1, 2, \cdots , n$) to decouple the $\textbf{U}$ and $\mathcal {W}$, we rewrite Eq. (6) with the following equivalent objective:

8 $$\begin{aligned}&\mathcal {J}^{ADMM}(\textbf{U},\textbf{F},\mathcal {W},\textbf{H}_0, \textbf{H}_1, \textbf{G}_0, \textbf{G}_1, \textbf{A}, \textbf{B}_{(2)}, \mathbf {\Lambda }_1, \mathbf {\Lambda }_{2,i}, \mathbf {\Lambda }_3, \mathbf {\Lambda }_{4,i}) =\nonumber \\&\text {tr}((\textbf{A}^{T}\textbf{G}_{1} - \textbf{F})^{T}\textbf{D}_{1}(\textbf{U}^{T}\textbf{G}_{1} - \textbf{F}))\nonumber \\&+ \gamma _1\sum _{i=1}^{n}\text {tr}((\textbf{X}_i-\textbf{W}_i\textbf{W}_i^T\textbf{X}_i)^T\textbf{D}_{2,i}(\textbf{X}_{i}-\textbf{B}_i\textbf{B}_i^T\textbf{X}_i))\nonumber \\&+\gamma _2\text {tr}((\mathcal {W}^T\otimes \textbf{X}-\textbf{H}_1\textbf{G}_1)^T\textbf{D}_3(\mathcal {W}^T\otimes \textbf{X}-\textbf{H}_1\textbf{G}_1))\nonumber \\&+ \gamma _3\text {tr}((\textbf{X}_{SNP}-\textbf{H}_0\textbf{G}_0)^T\textbf{D}_4(\textbf{X}_{SNP}-\textbf{H}_0\textbf{G}_0))\nonumber \\&+\gamma _4\text {tr}((\textbf{G}_1 - \textbf{G}_0)^T\textbf{D}_5(\textbf{G}_1 - \textbf{G}_0)) +\gamma _5\text {tr}(\textbf{H}_0^T\textbf{D}_6\textbf{H}_0)\nonumber \\&+\gamma _6\text {tr}(\textbf{W}^T_{(1)}\textbf{D}_7\textbf{W}_{(1)}) +\gamma _6\text {tr}(\textbf{W}^T_{(2)}\textbf{D}_8\textbf{B}_{(2)}) +\gamma _7\sum ^{c}_{q=1}(\textbf{a}_q^T\textbf{D}_{9,q}\textbf{a}_q)\nonumber \\&+\frac{\mu _1}{2}\left\| \textbf{F}_l-\textbf{Y}_l+\frac{1}{\mu _1}\mathbf {\Lambda }_1\right\| ^2_F +\sum _{i=1}^n\frac{\mu _{2,i}}{2}\left\| \textbf{W}^T_i\textbf{B}_i-\textbf{I}+\frac{1}{\mu _{2,i}}\mathbf {\Lambda }_{2,i}\right\| ^2_F\nonumber \\&+\frac{\mu _3}{2}\left\| \textbf{A} - \textbf{U} + \frac{1}{\mu _3}\mathbf {\Lambda }_3 \right\| ^2_F +\sum _{i=1}^n \frac{\mu _{4,i}}{2}\left\| \textbf{B}_i - \textbf{W}_i + \frac{1}{\mu _{4,i}} \mathbf {\Lambda }_{4,i} \right\| ^2_F, \end{aligned}$$

where $\mathbf {\Lambda }_1, \mathbf {\Lambda }_{2,i}, \mathbf {\Lambda }_3, \mathbf {\Lambda }_{4,i}$ are the Lagrangian multipliers for the constraints $\textbf{F}_l = \textbf{Y}_l,\ \textbf{W}_i^T \textbf{W}_i = \textbf{I},\ \textbf{A} = \textbf{U}, \text { and }\textbf{B}_i = \textbf{W}_i$. The detailed algorithm to minimize Eq. (8) is presented in Algorithm 1. In Algorithm 1, we use solution of Sylvester equation, such that $sylvester(\textbf{P}, \textbf{Q}, \textbf{R})$ gives an unique and exact solution for $\textbf{X}$ of equation $\textbf{P} \textbf{X} + \textbf{X} \textbf{Q} = \textbf{R}$. The time complexity of Algorithm 1 is $O(n r_1 d^2 (d + r_1))$ for each iteration where the step 11 is the most dominant. The detailed derivation of Algorithm 1 is provided in the Appendix.

**Figure:**
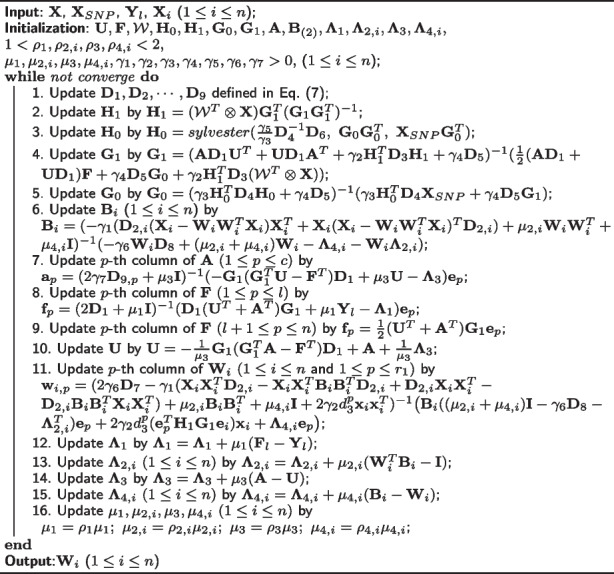
**Algorithm 1** Solve minimization problem in Eq. (8)

## Results

In this section, we introduce our experimental results about the clinical scores prediction task with the enriched biomarker representation and original biomarker representation to evaluate changes in the prediction performance from enrichment. Then we analyze the AD risk factors identified by the learned projections.

### Data preparation

We obtain the data used in our experiments from the ADNI database. We downloaded the MRI scans, SNP genotypes, and the longitudinal scores of Rey’s Auditory Verbal Learning Test (RAVLT) of 821 ADNI-1 participants. We perform voxel-based morphometry (VBM) and FreeSurfer automated parcellation on the MRI data as described by [12] and extract mean modulated gray matter (GM) measures for 90 target regions of interest (ROI). We follow SNP quality control steps discussed in [32]. Among 821 ADNI-1 participants, 412 participants are selected on the basis of existence of MRI records at Month 0/Month 6/Month 12/Month 24. Then we intentionally discard Month 24 scans with 50% probability to evaluate the learning capability of our model from longitudinal data with missing record. Our model learns the enrichment with the neuroimaging records from baseline to the second last visit, and project the last record (Month 12 or Month 24 with 50% probability) to predict the clinical scores at the last time point.

### Experimental settings

In our experiments, we aim to predict RAVLT clinical scores in the test set using two types of the inputs — the learned enriched representation and original representation of the most recent biomarkers. We use the different concatenations of SNPs, FS, and VBM modalities to assess the prediction performance of our model with diverse modalities. We split the dataset into a training and test set with a proportion of 80% and 20% each, therefore the number of participants is $l=323$ in the training set and $n-l = 89$ in the test set. The SNPs and MRI images of all *n* participants and clinical scores of only the *l* participants in training set are provided for our model to learn enriched representation. To predict the $n-l$ clinical scores in test set, we use the following conventional prediction models: Ridge linear Regression (RR), Convolutional Neural Network (CNN), and Support Vector Regression (SVR) which is the regression version of Support Vector Machine. We conduct a 5-fold cross-validation to search the set of best hyperparameters for each conventional model.

We conduct a 5-fold cross-validation to search the set of best hyperparameters for each conventional model. However the naive grid search can be time consuming especially when the combination of many hyperparameters is tuned. Instead of trying all the combinations of hyperparameters, we randomly choose the value from the grid of each hyperparameter. In order to increase the possibility of finding the better hyperparameters in the fewer searches, a randomly selected half of hyperparameters remain the best values found in the previous searches. In the 5-fold cross-validation, we search the best regularization parameter of RR in $\{10^3, 10^2, 10, 1, 10^{-1}, 10^{-2}, 10^{-3}\}$. For SVR, we fine tune the kernel function among sigmoid and radial basis function and box constraints in $\{10^3, 10^2, 10, 1, 10^{-1}, 10^{-2}, 10^{-3}\}$. We construct a 1-dimensional CNN configured as follows: (1) a convolutional layer with a window size of 5 $\times$ 16 (width $\times$ depth), followed by a rectified linear unit (ReLU) and a max pooling layer with a window size of 1 $\times$ 2; (2) a convolution layer with a window size of 10 $\times$ 32, followed by a ReLU and a max pooling layer with a window size of 1 $\times$ 2; (3) three fully connected layers where the number of nodes and dropout rate for each layer are fine tuned by searching the grid of $\{20, 60, 120\}$ and $\{0.3, 0.5, 0.7\}$ each. The hyperparameters of our enrichment model are tuned as following: $\gamma _1 = 10^{-1},\ \gamma _2 = 10^{-4},\ \gamma _3 = 10^{-2},\ \gamma _4 = 10^{-3},\ \gamma _5 = 10^{-1},\ \gamma _6 = 10^{-1},\ \gamma _7 = 10^{-1},\ \rho _1 = 1.05,\ \rho _2 = 1.05,\ \rho _3 = 1.15,\ \rho _4 = 1.15$.

### Experimental results

#### Original vs. enriched representation

**Fig. 3:**
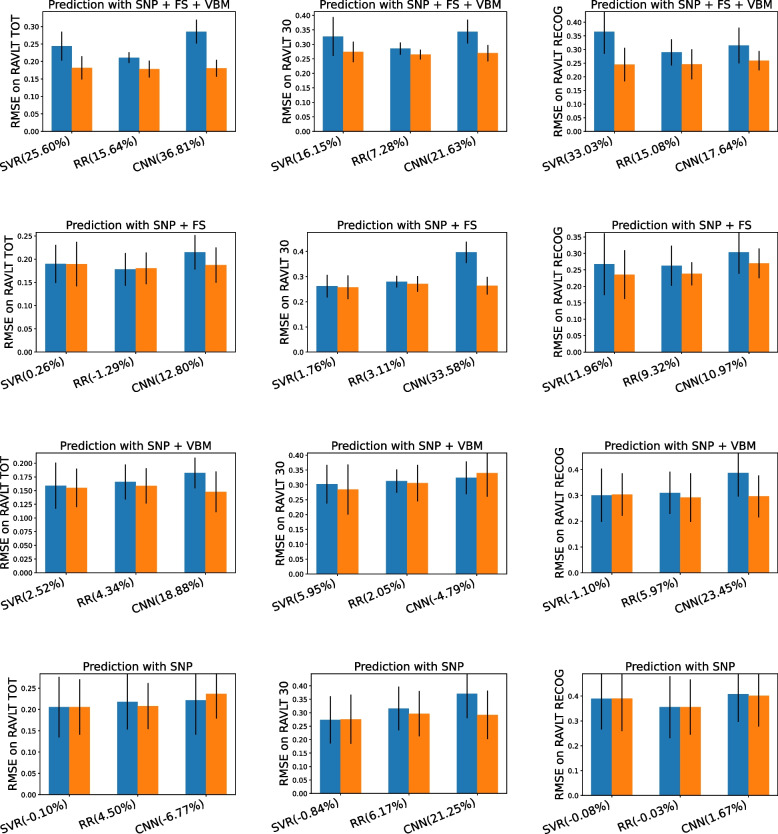
Comparison on prediction errors from original (blue) or enriched (orange) representation. The percentage % next to model name indicates the decreased amount in errors of predictions from the enriched representation. We also plot the standard deviation from 5-fold cross-validation at the head of each bar

In the experimental result reported in Fig. 3, we compute the Root Mean Squared Error (RMSE) between the ground truth clinical scores and predicted clinical scores from both the original and enriched representations. The result reveals that the prediction from enriched representation are mostly more accurate (9.84% in average) than the predictions supplied from the most recent record of original representation. Interestingly, among the various concatenation of modalities of biomarker measurements, the performance improvements of our enriched representation is larger when the many modalities are given. This indicates that our model fully utilizes the multi-modal dynamic data. Especially, the error gap is smallest when only SNPs are given. We suppose that this is because SNPs are static data which do not change along the time, genetic data is not able to provide the enough information about temporal variations of cognitive decline, while our model is designed to learn the temporal variations when dynamic data is given.

#### Identification of disease-relevant biomarkers

In addition to the cognitive outcomes prediction task, we identify AD relevant biomarkers using the weights of the learned projections. Since *p*-th feature in the enriched representation $\textbf{e}_p^T \textbf{W}^T_i \textbf{x}_i$ is weighted summation of the original biomarkers measurements, the weights summation in *q*-th row of the projection $\sum _{i=1}^n \left\| \textbf{e}_q^T \textbf{W}_i \right\| _1$ can be interpreted as AD relevance on the *q*-th biomarker.

##### Identified Neuroimaging Biomarkers

**Fig. 4:**
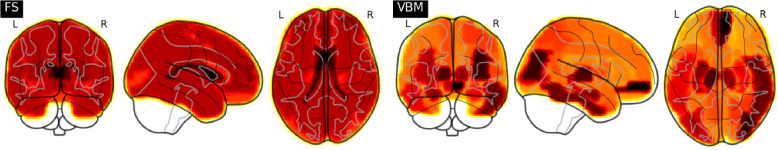
Visualization of weights distribution over the brain regions. The darker color indicates the larger weight on that region. The top four AD relevant regions are identified in FS: Right Caudate, Brodmann area 24, Left Thalamus, and Left Caudate, in VBM: Left Hippocampus, Left Amygdala, Left Thalamus, and Right Medial Orbito-frontal Cortex

In this aspect, we first identify the AD relevant imaging biomarkers, by plotting the weights of ROIs of VBM and FS in Fig. 4. These brain regions all appear in the medical literature associated with AD-related dimentia. For example, participants with cognitive decline showed atrophy of the caudate nucleus [33]. The volume of thalamus was significantly reduced [34] in participants diagnosed as probable AD. Furthermore, it has been found that the thalamus region plays an important role in generating attention, and anterior thalamus is in charge of declarative memory functioning [35]. The hippocampus is vulnerable to be damaged from AD [36] and has been shown to affect long-term memory and spatial navigation in participants with AD. Finally, the amygdala region, also identified by our approach, is also severely affected by AD [37] and is associated with emotional response and decision-making.

##### Identified Genotypic Biomarkers

**Fig. 5:**
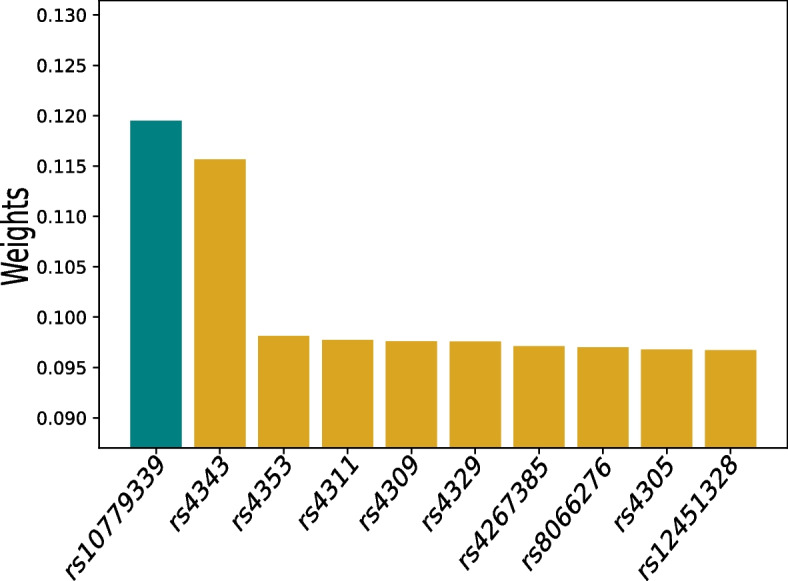
Weights on individual SNPs. The color of each bar denotes AlzGene group in Fig. 6

**Fig. 6:**
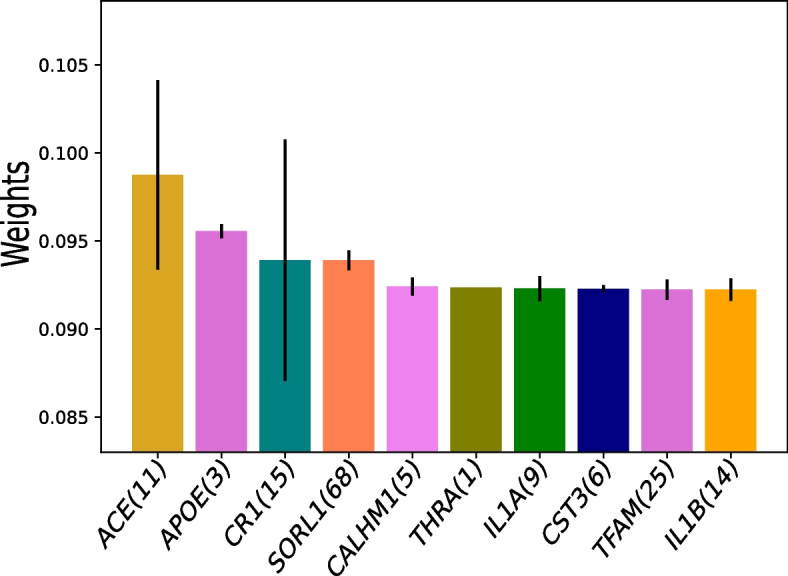
Weights on AlzGene groups. The number next to the AlzGene group name denotes the number of SNPs in that group

In addition, we identify AD relevant SNPs by plotting the weights on individual SNPs and AlzGene group of SNPs in Figs. 5 and 6. In Fig. 6, we download and use the AlzGene grouping information constructed by multiple genome-wide association studies listed on (http://www.alzgene.org/) [38]. The standard deviation of weights of SNPs in each AlzGene group is displayed as line length at the head of each bar. The top identified individual SNP, rs10779339, in Fig. 5 has been found to be related to cognitive decline [39]. Among the AlzGene groups in Fig. 6 the SNPs in the ACE (angiotensin-converting enzyme) group have been found to reduce the Amyloid Beta peptide (A$\beta$) which is commonly observed in the progression of AD-related cognitive decline [40]. Furthermore, the APOE (apolipoprotein E) gene, also identified by our approach is also involved in A$\beta$ aggregation and clearance [41].

In summary, the complex relationships between cognitive ability and identified biomarkers are clearly identified by our method and are well represented in previous AD research studies. This result supports the utility of our approach as a tool to discover and validate AD risk factors from multi-modal data.

## Discussion

Because our enrichment model incorporates the genetic and phenotypic biomarkers, it is possible to learn the enrichment from the different multi-modal data to predict the different target labels. From the experimental results in Fig. 3, the prediction performance is further improved as the many modalities of data are given. For example, Diffusion Tensor Imaging (DTI) is an effective tool to investigate the white matter organization of the brain and AD progression [42]. Compared to MRI, blood based biomarkers can be measured less costly and intrusive and A$\beta$ levels in blood can aid in the early diagnosis of AD [43]. We can learn the projections for these additional modalities, and concatenate the projections in Eq. (2) and Eq. (5). While considering the complexity of Algorithm 1 proportional to $d^3$ and the increasing dimensionality *d* with the many modalities of measurements, our model can be flexibly applied to the diverse datasets and prediction tasks.

## Conclusion

Missing data is a major issue in longitudinal multi-modal healthcare datasets. This research aims to devise a novel methodology to learn a consistent length representation for all the participants in the ADNI dataset. The learned biomarker representation summarizes the genetic biomarkers and their group structure, known clinical scores, and all the available records of longitudinal biomarkers on a per-participant basis. Our experiments show that the learned enriched representation outperforms the original measurement in predicting the clinical scores. Finally, the identified AD relevant biomarkers are in nice accordance with existing research findings, indicating the utility of our approach.

## Figures and Tables

**Figure:**
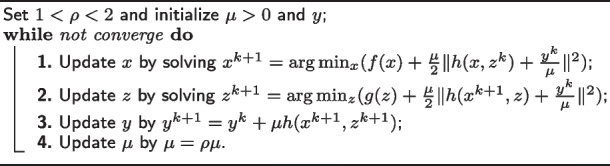
**Algorithm 2** The ADMM algorithm

## Data Availability

The data used in this work are from public datasets: ADNI (http://adni.loni.usc.edu/). The access to these datasets is managed through secure LONI image and data archive (https://ida.loni.usc.edu/login.jsp) and contingent on adherence to the ADNI data use agreement and the publications’ policies. To apply for the access to data please visit: http://adni.loni.usc.edu/data-samples/access-data/.

## Appendix

In this section, we show how Algorithm 1 is derived from our smoothed objective Eq. (6). ADMM solves convex optimization problems by breaking them into smaller pieces that are easier to handle. Specifically, given the following objective with the equality constraint:

9 $$\begin{aligned} \min _{x,z} f(x)+g(z),\qquad s.t.\quad h(x,z)=0, \end{aligned}$$

Algorithm 2 solves the problem in Eq. (9) by decoupling it into subproblems and optimizing each variable while fixing others, where *y* is the Lagrangian multiplier to the constraint *h*. We extend Algorithm 2 of two variables to Algorithm 1 of the set of variables $\{\textbf{U},\textbf{F},\mathcal {W},\textbf{H}_0, \textbf{H}_1, \textbf{G}_0, \textbf{G}_1, \textbf{A}, \textbf{B}_{(2)}\}$. To be specific, we earn a update equation respect to each variable in Algorithm 1, by taking the derivation over a each variable and set it to zero matrix, while fixing other variables. For the step 2 in Algorithm 1, the derivative is:

10 $$\begin{aligned} \frac{\partial \mathcal {J}^{ADMM}}{\partial \textbf{H}_1} = -2 \gamma _2 \textbf{D}_3 (\mathcal {W} \otimes \textbf{X} - \textbf{H}_1 \textbf{G}_1) \textbf{G}_1^T. \end{aligned}$$

For the step 3, the derivative is:

11 $$\begin{aligned} \frac{\partial \mathcal {J}^{ADMM}}{\partial \textbf{H}_0} = - 2 \gamma _3 \textbf{D}_4 (\textbf{X}_{SNP} - \textbf{H}_0 \textbf{G}_0) \textbf{G}_0^T + 2 \gamma _5 \textbf{D}_6 \textbf{H}_0. \end{aligned}$$

For the step 4, the derivative is:

12 $$\begin{aligned}&\frac{\partial \mathcal {J}^{ADMM}}{\partial \textbf{G}_1} = \textbf{A} \textbf{D}_1 (\textbf{U}^T \textbf{G}_1 - \textbf{F}) + \textbf{U} \textbf{D}_1 (\textbf{A}^T \textbf{G}_1 - \textbf{F})\nonumber \\&+ 2 \gamma _4 \textbf{D}_5 (\textbf{G}_1 - \textbf{G}_0) - \gamma _2 \textbf{H}_1^T \textbf{D}_3 (\mathcal {W}^T \otimes \textbf{X} - \textbf{H}_1 \textbf{G}_1). \end{aligned}$$

For the step 5, the derivative is:

13 $$\begin{aligned} \frac{\partial \mathcal {J}^{ADMM}}{\partial \textbf{G}_0} = - 2 \gamma _3 \textbf{H}_0^T \textbf{D}_4 (\textbf{X}_{SNP} - \textbf{H}_0 \textbf{G}_0) - 2 \gamma _4 \textbf{D}_5 (\textbf{G}_1 - \textbf{G}_0). \end{aligned}$$

For the step 6, the derivative is:

14 $$\begin{aligned}&\frac{\partial \mathcal {J}^{ADMM}}{\partial \textbf{B}_i} = \gamma _6 \textbf{W}_i \textbf{D}_8\nonumber \\&- \gamma _1 (\textbf{D}_{2,i}(\textbf{X}_i - \textbf{W}_i \textbf{W}_i^T \textbf{X}_i^T) \textbf{X}_i^T + (\textbf{D}_{2,i}(\textbf{X}_i - \textbf{W}_i \textbf{W}_i^T \textbf{X}_i^T) \textbf{X}_i^T)^T)\textbf{B}_i\nonumber \\&+ \mu _{2,i} \textbf{W}_i \textbf{W}_i^T \textbf{B}_i - \mu _{2,i} \textbf{W}_i + \textbf{W}_i \mathbf {\Lambda }_{2,i} + \mu _{4,i} (\textbf{B}_i - \textbf{W}_i + \frac{1}{\mu _{4,i}} \mathbf {\Lambda }_{4,i}). \end{aligned}$$

For the step 7 ($1 \le p \le c$), the derivative is:

15 $$\begin{aligned} \frac{\partial \mathcal {J}^{ADMM}}{\partial \textbf{a}_p} = (\textbf{G}_1 (\textbf{G}_1^T \textbf{U} - \textbf{F}^T) \textbf{D}_1 - \mu _3 \textbf{U} + \mathbf {\Lambda }_3 )\textbf{e}_p + 2 \gamma _7 \textbf{D}_{9,i} \textbf{a}_p + \mu _3 \textbf{a}_p. \end{aligned}$$

For the step 8 ($1 \le p \le l$), the derivative is:

16 $$\begin{aligned} \frac{\partial \mathcal {J}^{ADMM}}{\partial \textbf{f}_p} = \big (\textbf{D}_1 (\textbf{U}^T \textbf{G}_1 - \textbf{F}) + \textbf{D}_1 (\textbf{A}^T \textbf{G}_1 - \textbf{F}) + \mu _1 (\textbf{F} - \textbf{Y} + \frac{1}{\mu _1} \mathbf {\Lambda }_1)\big )\textbf{e}_p. \end{aligned}$$

For the step 9 ($l + 1 \le p \le n$), the derivative is:

17 $$\begin{aligned} \frac{\partial \mathcal {J}^{ADMM}}{\partial \textbf{f}_p} = (\textbf{D}_1 (\textbf{U}^T \textbf{G}_1 - \textbf{F}) + \textbf{D}_1 (\textbf{A}^T \textbf{G}_1 - \textbf{F}))\textbf{e}_p. \end{aligned}$$

For the step 10, the derivative is:

18 $$\begin{aligned} \frac{\partial \mathcal {J}^{ADMM}}{\partial \textbf{U}} = \textbf{G}_1 (\textbf{G}_1^T \textbf{A} - \textbf{F}^T) \textbf{D}_1 + \mu _3 (\textbf{A} - \textbf{U} + \frac{1}{\mu _3} \mathbf {\Lambda }_3). \end{aligned}$$

For the step 11 ($1 \le p \le r_1$), the derivative is:

19 $$\begin{aligned}&\frac{\partial \mathcal {J}^{ADMM}}{\partial \textbf{w}_{i,p}} =\nonumber \\&\big (- \gamma _1 (\textbf{X}_i \textbf{X}_i^T \textbf{D}_{2,i} - \textbf{X}_i \textbf{X}_i^T \textbf{B}_i \textbf{B}_i^T \textbf{D}_{2,i} + \textbf{D}_{2,i} \textbf{X}_i \textbf{X}_i^T - \textbf{D}_{2,i} \textbf{B}_i \textbf{B}_i^T \textbf{X}_i \textbf{X}_i^T) \textbf{W}_i\nonumber \\&+ \gamma _6 \textbf{B}_i \textbf{D}_8 + 2 \gamma _6 \textbf{D}_7 \textbf{W}_i + \mu _{2, i} \textbf{B}_i \textbf{B}_i^T \textbf{W}_i - \mu _{2, i} \textbf{B}_i + \textbf{B}_i \mathbf {\Lambda }^T_{2,i} - \mu _{4,i} \textbf{B}_i\nonumber \\&+ \mu _{4, i} \textbf{W}_i - \mathbf {\Lambda }_{4,i}\big )\textbf{e}_i + 2 \gamma _2 d_3^p \textbf{x}_i (\textbf{x}_i^T \textbf{w}_{i,p} - \textbf{e}_p^T \textbf{H}_1 \textbf{G}_1 \textbf{e}_i). \end{aligned}$$

By setting the derivative of each step to zero matrix, the update equations in Algorithm 1 can be obtained.

## Abbreviations

AD: Alzheimer’s disease
MRI: Magnetic resonance imaging
FS: FreeSurfer
VBM: Voxel-based morphometry
SNP: Single-nucleotide polymorphism
RAVLT: Ray’s auditory verbal learning test
ADNI: Alzheimer’s disease neuroimaging initiative
PCA: principal component analysis
ADMM: Alternating direction method of multipliers
RR: Ridge linear regression
CNN: Convolutional neural network
SVR: Support vector regression
RMSE: Root mean squared error
ReLU: Rectified linear unit
